# Physiological demands and signaling associated with snake venom production and storage illustrated by transcriptional analyses of venom glands

**DOI:** 10.1038/s41598-020-75048-y

**Published:** 2020-10-22

**Authors:** Blair W. Perry, Drew R. Schield, Aundrea K. Westfall, Stephen P. Mackessy, Todd A. Castoe

**Affiliations:** 1 Department of Biology, The University of Texas Arlington, 501 S. Nedderman Dr., Arlington, TX 76019 USA; 2 Department of Ecology and Evolutionary Biology, University of Colorado, Boulder, CO 80309 USA; 3 School of Biological Sciences, University of Northern Colorado, 501 20th Street, Greeley, CO 80639 USA

**Keywords:** Gene expression, Physiology

## Abstract

Despite the extensive body of research on snake venom, many facets of snake venom systems, such as the physiology and regulation of the venom gland itself, remain virtually unstudied. Here, we use time series gene expression analyses of the rattlesnake venom gland in comparison with several non-venom tissues to characterize physiological and cellular processes associated with venom production and to highlight key distinctions of venom gland cellular and physiological function. We find consistent evidence for activation of stress response pathways in the venom gland, suggesting that mitigation of cellular stress is a crucial component of venom production. Additionally, we demonstrate evidence for an unappreciated degree of cellular and secretory activity in the steady state venom gland relative to other secretory tissues and identify vacuolar ATPases as the likely mechanisms driving acidification of the venom gland lumen during venom production and storage.

## Introduction

The snake venom gland is an intriguing yet poorly understood system that holds broad potential as a model for studying the evolution of novel organ function and regulatory architecture, cellular responses to extreme physiological demands, and the production and storage of potent biological toxins. The emerging interest and potential utility of this system is emphasized by the recent development of snake venom gland organoids, an unprecedented resource for the controlled study of snake venom regulation, production, and general snake venom gland physiology and function^1^. However, despite an extensive body of literature focused on the products of snake venom glands (i.e. venoms and their components), and to a lesser degree the genes underlying snake venoms, little is known about the physiological, cellular, and molecular functionality of snake venom glands and how these compare to other secretory systems. The lack of a systems-level understanding of snake venom gland biology presents a major impediment to progress towards a comprehensive understanding of venom system evolution and function.

Our current understanding of venom gland function and physiology indicates that the process of venom production is impressively dynamic. Following the depletion of stored venom (i.e. via a predatory bite or manual venom extraction), the snake venom gland exhibits rapid and high-magnitude upregulation of venom gene transcription, venom protein production and processing, and secretion of venom components into the gland lumen^2–7^. Aspects of this process have been described through microscopy, proteomic, and gene expression^2,5,8–11^, including some broad characterization of non-venom gene expression^3,12^. While these studies have provided insight into venom gland function, their focus has been on processes immediately associated with venom production (i.e. regulation of venom genes and secretion of venom components). Accordingly, a systems-level understanding of the cellular processes that comprise the physiological environment in which venom production occurs remains incompletely characterized. For example, the high demands of gene regulation, protein processing and venom protein production places extreme demands and stress on venom gland epithelial cells. This would necessitate the activation of cellular stress response mechanisms to facilitate successful venom production while preventing damage to protein products, cells or the venom gland tissue. It is therefore expected that the extreme demands placed on venom gland tissue during venom production are associated with similarly extreme cellular physiology to accommodate extreme cellular and physiological performance, yet this has not been examined in previous studies.

In addition to unique physiology and functionality associated with the upregulation of venom production following venom depletion, the steady-state venom gland is tasked with housing an abundance of highly toxic venom components in a manner that protects the venom gland and surrounding tissue from the biological activity of venom components while keeping stored venom stable. Previous studies have shown that acidification of the venom bolus in the gland lumen inhibits venom enzymatic activity during storage and thus plays an important role in self-protection against harmful effects of venom and stabilization of venom proteins^9^. It has been proposed that this acidification is driven by populations of mitochondria-rich cells with morphological and histochemical features similar to parietal cells in mammals, which are responsible for secretion of gastric acid^9^. However, the exact molecular mechanisms underlying venom gland acidification remain unexplored.

In this study, we use gene expression data from multiple sampled timepoints from the venom gland of the Prairie Rattlesnake (*Crotalus viridis*) to facilitate the first detailed analysis of physiological and cellular pathways associated with the rapid and high-magnitude shifts in activity and function of the snake venom gland during venom production. These analyses include both broad characterization of regulatory pathways, molecules, and analysis of differentially expressed genes, as well as targeted dissection of cellular stress response mechanisms that may play an underappreciated role facilitating venom production. We also conduct comparisons of venom gland gene expression to that of multiple non-venom secretory tissues to highlight physiological and functional distinctiveness of the venom gland, including detailed analysis of molecular mechanisms driving venom gland lumen acidification.

## Methods

Tissue samples for unextracted, one and three days post-extraction (DPE) venom gland, as well as skin, pancreas, and stomach were sampled as part of a previous study on the genome of the Prairie Rattlesnake^3^. All animal work was conducted with approved and registered IACUC protocols (University of Northern Colorado, 1701D-SM-S-20). Using these tissue resources, we generated new poly-A selected mRNA libraries for this study and sequenced these on an Illumina NovaSeq using 150 bp paired-end reads. Raw RNAseq data was quality-trimmed and filtered using Trimmomatic v0.33^13^ and mapped to the Prairie Rattlesnake reference genome using STAR v2.5.2b^14^. Raw read counts were generated with featureCounts v1.6.3^15^. Count normalization and pairwise comparisons between unextracted and 1DPE venom gland, 1DPE and 3DPE venom gland, and body tissue and unextracted venom gland were conducted in DeSeq2 v1.26.0^16^ with independent hypothesis weighting (IHW) *p*-value correction^17^. Differentially expressed genes were defined as those with IHW *p*-value < 0.05.

Differentially expressed genes not previously annotated as venom genes were assigned an orthologous human gene identifier using orthology tables generated previously^18^ and analyzed using Core Analysis in Ingenuity Pathway Analysis (IPA)^19^. IPA inferences of canonical pathway and upstream regulatory molecule activity with an overlap *p*-value < 0.05 and absolute activation z-score > 1 were considered significant. Gene ontology (GO) analyses were performed specifically on sets of genes upregulated in the unextracted venom gland relative to non-venom tissues, and for those upregulated in 1DPE relative to unextracted venom gland tissues using the ClueGO plugin v2.5.6^20^ for Cytoscape v3.7.2^21^. GO analysis of 1DPE versus 3DPE were not conducted due to the low number of genes differentially expressed between these time points.

Expression for set of candidate genes annotated with the “pH reduction” GO term (GO: 0045851) were analyzed to identify putative mechanisms of venom gland acidification, and inferences derived from gene expression data were validated using a combination of Western blot and immunohistochemistry staining of gastric and venom gland membranes from *C. viridis*. Additional detail for all methods is provided in the Supplementary Information Methods. We note that original, uncropped images of gels were regrettably lost and are therefore not shown. However, we declare that no adjustments or manipulations were made the raw gel images besides cropping to emphasize focal bands.

## Results

### Timing and variation in snake venom gene expression

**Figure 1:**
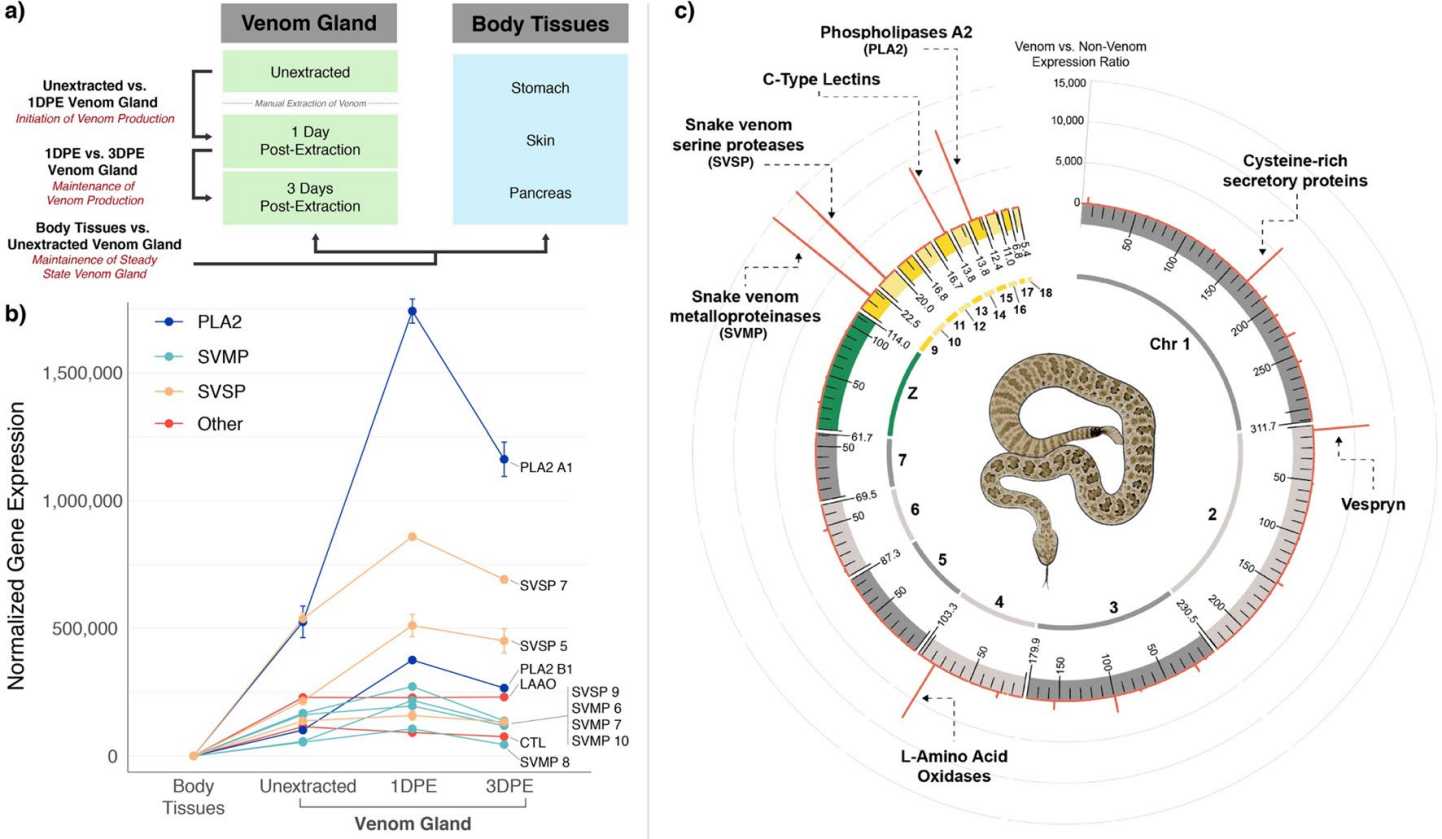
Experimental design and venom gene expression. (**a**) Overview of experimental design, highlighting the three pairwise comparisons used to characterize features of venom gland physiology. (**b**) Expression of major venom genes across sampled timepoints and in non-venom tissues. (**c**) Genome-wide view of gene expression in the rattlesnake venom gland at one day post-extraction (1DPE), with the red line indicating the ratio of expression between 1DPE venom gland and non-venom tissues. Major venom gene clusters (labeled) are easily distinguishable given their high magnitude of expression.

Because previous gene expression studies on venom glands have primarily focused specifically on expression of venom genes, we first analyzed patterns of venom gene expression to illustrate the degree of upregulation of venom gene production following venom depletion. At 1DPE, average expression of major venom genes is significantly upregulated (Fig. 1b) to the extent that venom gene expression dwarfs that of non-venom genes at a genome-wide scale (Fig. 1c). For nearly all of these venom genes, expression decreases between 1 and 3DPE, although expression remains elevated during this interval relative to the expression in unextracted venom gland for most venom genes (Fig. 1b). In contrast, venom genes are not expressed at notable levels in the three sampled body tissues (Fig. 1b).

### Differential expression of all non-venom genes across time points

**Figure 2:**
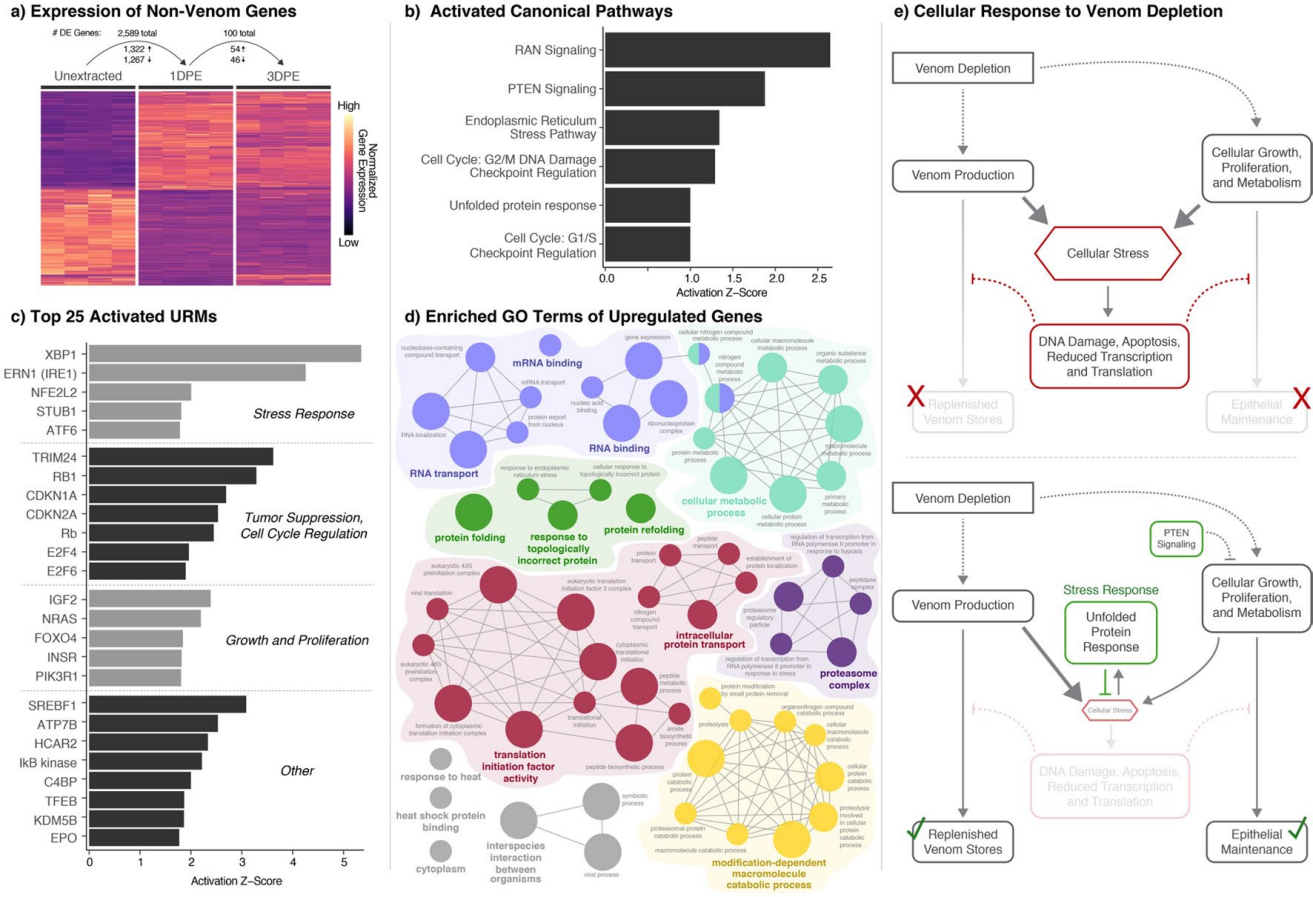
Functional characterization of gene expression in the venom gland during venom production. (**a**) Gene expression heatmap of all genes showing significant differential expression between unextracted versus 1DPE and 1DPE vs. 3DPE pairwise comparisons. The total number of differentially expressed (DE) genes, as well as the number up- and downregulated, in each comparison is shown above the heatmap. (**b**) Inferences of activated canonical pathways based on differentially expressed genes between unextracted and 1DPE venom gland tissues. **c**) Inferences of activated upstream regulatory molecules (URMs) based on differentially expressed genes between unextracted and 1DPE venom gland tissues, with post-hoc categorization based on known activity of URMs. (**d**) Significantly enriched GO terms of genes significantly upregulated at 1DPE. All terms shown are significantly enriched (*p* < 0.05), and node size is inversely proportional to enrichment *p*-value, with larger nodes having a lower *p*-value. Connected nodes indicate a high proportion of shared genes underlying term enrichment. (**e**) Overview of proposed roles of stress response activation in facilitating venom production and epithelial maintenance in the venom gland.

To characterize the full cellular and physiological response of the venom gland during venom production, we focused on analyses of differentially expressed genes that are not known venom genes (Fig. 2). Between the unextracted venom gland and 1 DPE venom gland samples, we identified 2589 differentially expressed non-venom genes, roughly half of which are upregulated (1322 genes). Comparatively few genes are differentially regulated between 1 and 3 DPE, with only 100 genes differentially expressed, 54 of which were upregulated (Fig. 2a). Given the small number of differentially expressed genes between 1 and 3DPE, we focused on the unextracted versus 1DPE and unextracted versus non-venom tissues gene sets in downstream functional inferences.

### Regulatory mechanisms associated with venom gland physiology during venom production

To characterize molecular mechanisms involved in gland physiology during venom production, we performed Core Analysis in Ingenuity Pathway Analysis on the 2589 genes that showed significant differential expression between unextracted and 1DPE venom gland tissue (Fig. 2a–c). Core Analysis separately infers relative changes in regulatory activity of both canonical pathways and upstream regulatory molecules (URMs) based on observed patterns of differential gene expression. We separately performed GO term overrepresentation analysis to identify functional categories of genes that were enriched in genes that were upregulated during venom production (Fig. 2d). Together, these analyses highlight three distinct categories of molecular regulatory activity in the venom gland during venom regulation (Fig. 2).

All functional analyses of gene expression during venom production provide evidence for activation of stress response mechanisms (Fig. 2b–d). Four of the six canonical pathways inferred to have increased activity at 1DPE are stress response pathways. These include the endoplasmic reticulum stress response and unfolded protein response pathways, which are associated with mitigating cellular stress caused by misfolded proteins and high demands for protein processing, as well as two DNA damage checkpoint regulation pathways that act to prevent replication of cells that have accumulated significant DNA damage (Fig. 2b). Activation of stress response mechanisms is also emphasized in inferences of URM activity, in which endoplasmic reticulum to nucleus signaling 1 (ERN1, a.k.a. IRE1) and activating transcription factor 6 (ATF6), two of the three primary stress sensors that lead to activation of the unfolded protein response, are inferred to be activated (Fig. 2c). Further, x-box binding protein 1 (XBP1), a transcription factor activated by IRE1 in response to ER stress that subsequently upregulates genes associated with protein folding and degradation, is inferred to have the highest increase in activity among URMs between unextracted and 1DPE venom gland samples (Fig. 2c). Two additional stress related URMs are inferred as activated during venom production, including nuclear factor erythroid 2 like 2 (NFE2L2*,* a.k.a. NRF2), a high-level regulator of the NRF2 oxidative stress response pathway, and STIP1 homology and u-box containing protein 1 (STUB1), which targets misfolded proteins for degradation (Fig. 2c). Similarly, GO term analysis of significantly upregulated genes between unextracted and 1DPE show overrepresentation of several terms related to cellular stress responses, including “response to topologically incorrect protein” and terms related to the proteasome complex (Fig. 2d).

Multiple pathways and URMs related to cellular growth and proliferation, cell cycle regulation and tumor suppression are also inferred to be activated during venom regulation (Fig. 2b,c). The PTEN signaling pathway, which negatively regulates cell growth and proliferation, increases significantly in activity at 1DPE (Fig. 2b), along with URMs involved in regulation of cellular growth and proliferation, including insulin like growth factor 2 (IGF2), insulin receptor (INSR) and phosphoinositide-3-kinase regulatory subunit 1 (PIK3R1; Fig. 2c). Additional URMs with high estimated increases in activity during venom production include sterol regulatory element binding transcription factor 1 (SREBF1) and hydrocarboxylic acid receptor 2 (HCAR2), which are involved in lipid signaling and metabolism; ATPase copper transporting beta (ATP7B), IκB kinase, an upstream regulator within the Nf-κB signaling pathway; and transcription factor EB (TFEB), a regulator of lysosome biogenesis and pro-autophagy signaling, among others (Fig. 2c).

We also find broad evidence of pathways, regulators and functional groups of genes associated with the pronounced secretory function of the venom gland during venom production. These include the RAN signaling pathway, which is inferred to have the highest degree of activation at 1DPE (Fig. 2b) and is involved with nuclear transport of proteins and RNAs, as well as numerous enriched GO term categories associated with the processes of transcription, translation, protein transport, modification, and metabolism (Fig. 2d).

### Steady-state regulatory mechanisms that differentiate the venom gland from other tissues

**Figure 3:**
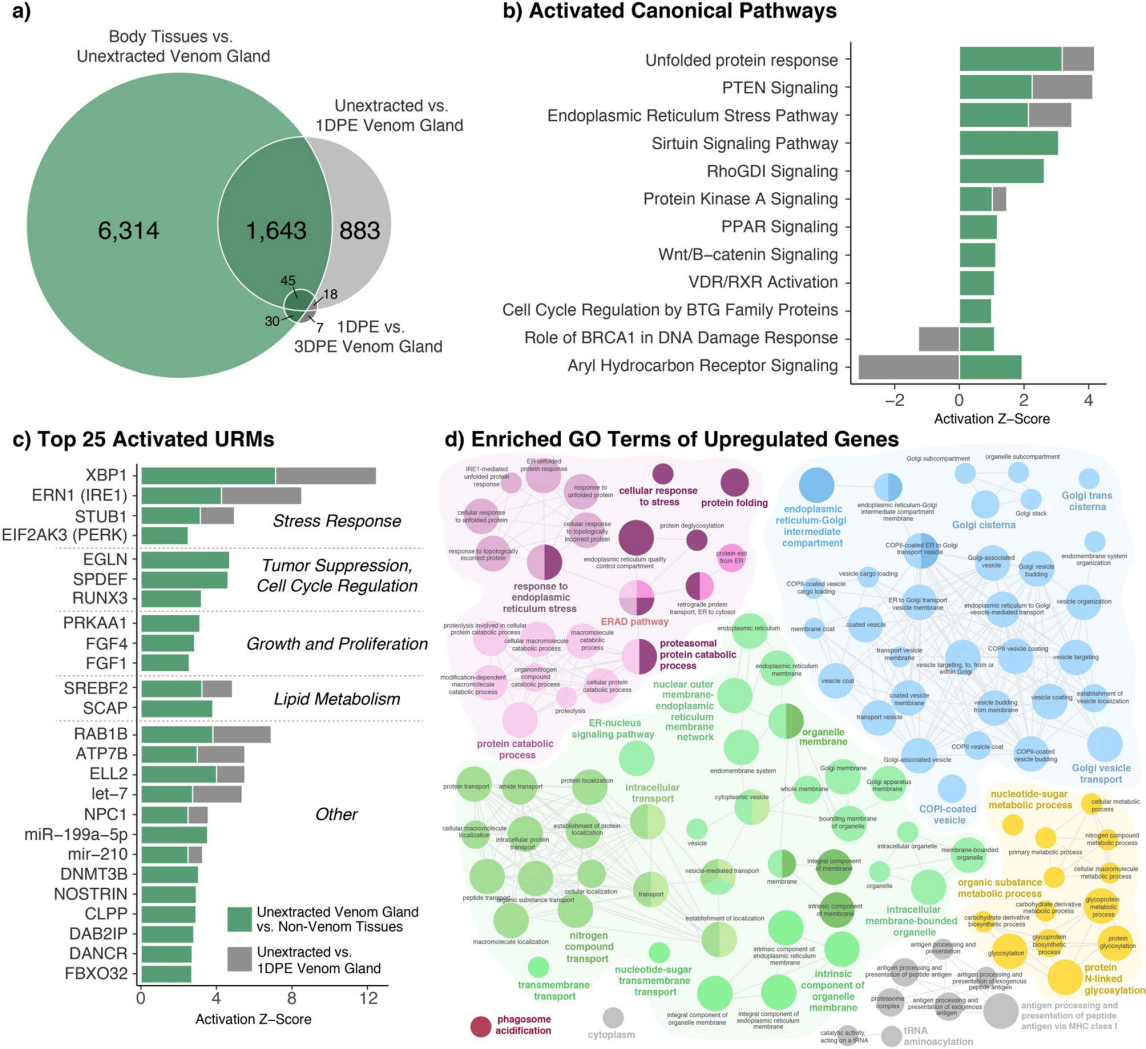
Functional characterization of gene expression in the venom gland relative to other non-venom secretory tissues. (**a**) Venn diagram denoting shared and unique genes between all pairwise comparisons. (**b**) Inferences of activated canonical pathways based on differentially expressed genes between non-venom tissues and the unextracted venom gland (green), with inferred activity in analyses of unextracted versus 1DPE venom gland tissues shown in grey if present. (**c**) Top 25 inferences of activated URMs based on differentially expressed genes between non-venom tissues and the unextracted venom gland (green), with inferred activity in analyses of unextracted versus 1DPE venom gland tissues shown in grey if present. (**d**) Significantly enriched GO terms of genes significantly upregulated in the unextracted venom gland relative to body tissues. All terms shown are significantly enriched (*p* < 0.05), and node size is inversely proportional to enrichment *p*-value, with larger nodes having a lower *p*-value. Connected nodes indicate a high proportion of shared genes underlying term enrichment.

To characterize the physiological and regulatory distinctiveness of the venom gland at a steady state, we identified regulatory mechanisms and enriched categories of differentially expressed genes from comparisons between the unextracted venom gland and a group of other secretory organs (pancreas, skin, and stomach), and compared these to inferences of regulatory activity during venom production (Fig. 3). We identified 8032 significantly differentially expressed genes, of which 3134 are upregulated, in the unextracted venom gland tissue relative to body tissues (Fig. 3a). A total of 1688 differentially expressed genes are identified both in pairwise comparisons of unextracted venom gland to body tissues and unextracted venom gland to 1DPE venom gland (Fig. 3a).

This analysis showed evidence for high activity of cellular stress response mechanisms in the unextracted venom gland relative to non-venom secretory tissues (Fig. 3b–d). The unfolded protein response and endoplasmic reticulum stress pathways, both of which showed evidence of activation during venom production, are here inferred to be activated to a greater degree in the unextracted venom gland relative to body tissues (Fig. 3b), indicating that these pathways exhibit a high baseline activity in the steady-state venom gland and are further upregulated during venom production. Similar to inferences during venom production, XBP1 and ERN1 (IRE1) are inferred to be relatively active in the venom gland compared to non-venom body tissues, as well as eukaryotic translation initiation factor 2 alpha kinase 3 (EIF2AK3, a.k.a. PERK), another high-level regulator within the unfolded protein response (Fig. 3c). Overrepresented GO terms include “response to endoplasmic reticulum stress” and other terms related to responses to misfolded proteins (Fig. 3d), indicating a relatively high degree of endoplasmic reticulum stress in the unextracted venom gland relative to other secretory tissues.

Several pathways and URMs involved in tumor suppression, cell cycle regulation, and regulation of cellular growth and proliferation are relatively highly active in the unextracted venom gland, and these are largely exclusive of regulatory mechanisms implicated in venom production. For example, while PTEN is inferred to be upregulated both in this analysis and during venom production, pathways including protein kinase A signaling, PPAR signaling and Wnt/β-catenin signaling pathways are uniquely activated in the unextracted venom gland (Fig. 3b). Further, while multiple URMs broadly involved in insulin-related growth signaling (i.e. IGF2, INSR, and PI3KR1) show evidence of activation during venom production, these URMs are not observed in the unextracted venom gland and instead two fibroblast growth factor (FGF) transcription factors show evidence of activation. Additional URMs with inferred activation in the unextracted venom gland include Ras-related protein Rab-1B (RAB1B), a regulator of intracellular vesicle transport between the ER and Golgi, which is also activated during venom production (Fig. 3c).

Analyses of GO terms identified a substantially greater number of overrepresented terms in this analysis compared to those during venom production that were directly associated with secretory machinery of the venom gland, including many associated with the activity of the endoplasmic reticulum and Golgi, intracellular transport via vesicles, and protein folding, export, and metabolism (Figs. 2d, 3d). In contrast, fewer terms associated with regulation of transcription and translation are overrepresented in this set of genes upregulated in the unextracted venom gland relative to other secretory tissues (Figs. 2d, 3d).

### Detailed analysis of unfolded protein response activity in the snake venom gland

**Figure 4:**
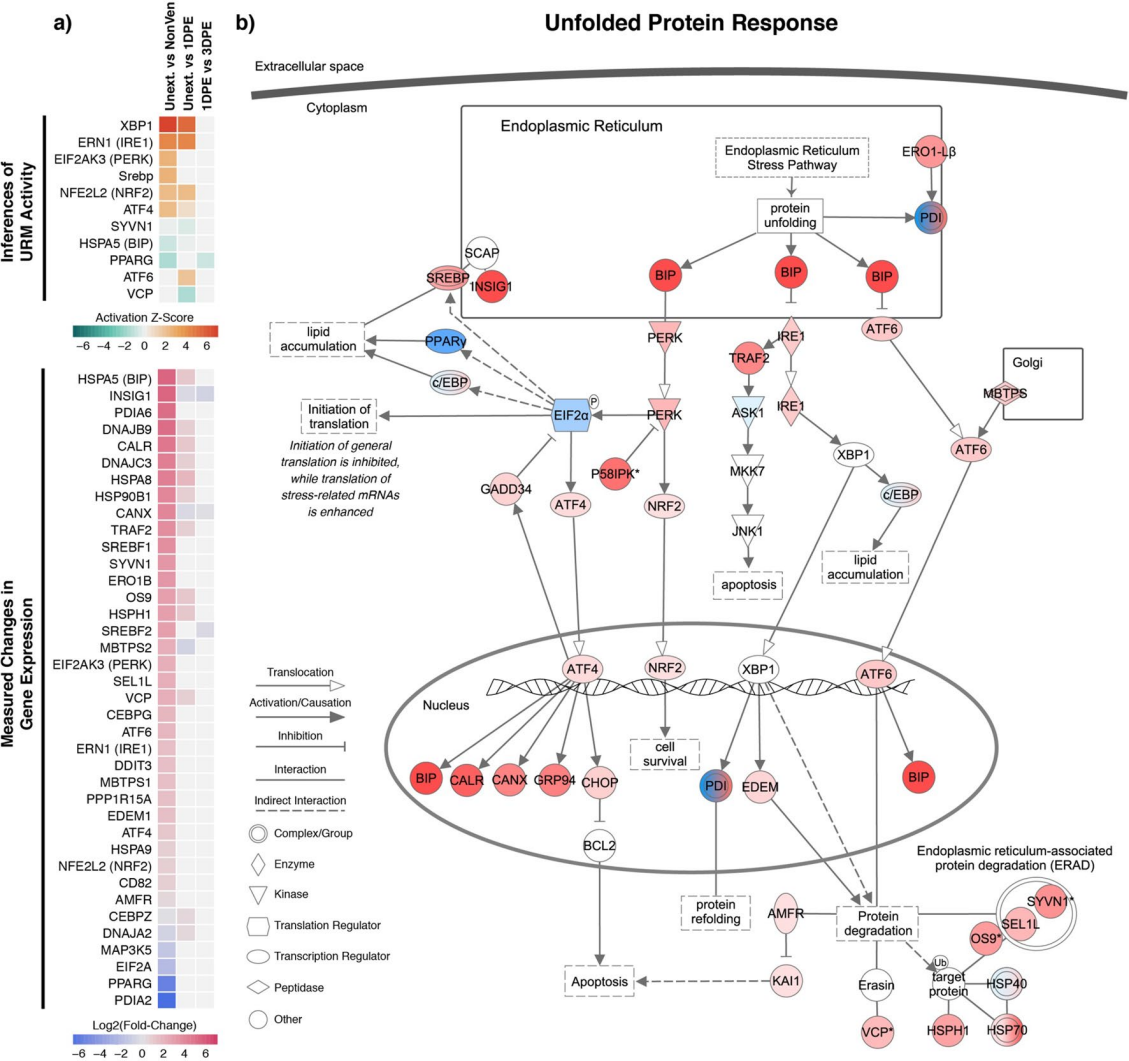
Details of unfolded protein response activation in the venom gland. (**a**) heatmap of inferred upstream regulatory molecule activity (top) and observed differential expression of genes involved in the unfolded protein response (bottom), (**b**) Diagram of the unfolded protein response pathway overlaid with observed patterns of differential gene expression in the unextracted venom gland relative to other non-venom tissues, with red indicating upregulation, blue indicating downregulation, and darker colors indicating a higher magnitude change in expression.

Our analyses of the venom gland both in an unextracted and post-extracted state consistently infer activation of stress response mechanisms, and primarily those of the unfolded protein response (UPR; Figs. 2, 3). To better understand the activation of this pathway, we characterized all genes, URMs, and specific components of the UPR pathway that are activated in the venom gland (Fig. 4). Pairwise comparisons between the unextracted venom gland and body tissues yielded the largest number of differentially expressed genes involved with the unfolded protein response pathway, the majority of which are upregulated in the venom gland relative to other secretory tissues (Fig. 4a). A subset of these genes is further differentially expressed at 1DPE with the majority upregulated, and only three are differentially expressed between 1 and 3DPE (Fig. 4a). Notably, all three of the primary high-level regulators of the UPR—*PERK* (*EIF2AK3*), IRE1 (ERN1), and *ATF6*—are upregulated in the unextracted venom gland relative to other secretory tissues (Fig. 4a,b). These analyses also suggest activation of the NRF2 oxidative stress response pathway in unextracted and 1DPE venom gland tissue given the inferred activation of its primary regulator, NFE2L2 (Fig. 4).

Consistent with the prevalence of overrepresented GO terms related to ER stress response and protein degradation, many genes involved in endoplasmic reticulum associated protein degradation (ERAD) are significantly upregulated in the venom gland (Fig. 4b). Additionally, both the inferred activation of SREBP transcription factor as a URM as well as the observed upregulation of its constituent genes (SREBF1 and SREBF2) indicate potential crosstalk between the UPR and lipid signaling mechanisms and pathways (such as PPAR signaling) that are separately inferred to exhibit unique patterns of activity in the venom gland (Fig. 4b).

### Candidate gene analysis of venom gland acidification

**Figure 5:**
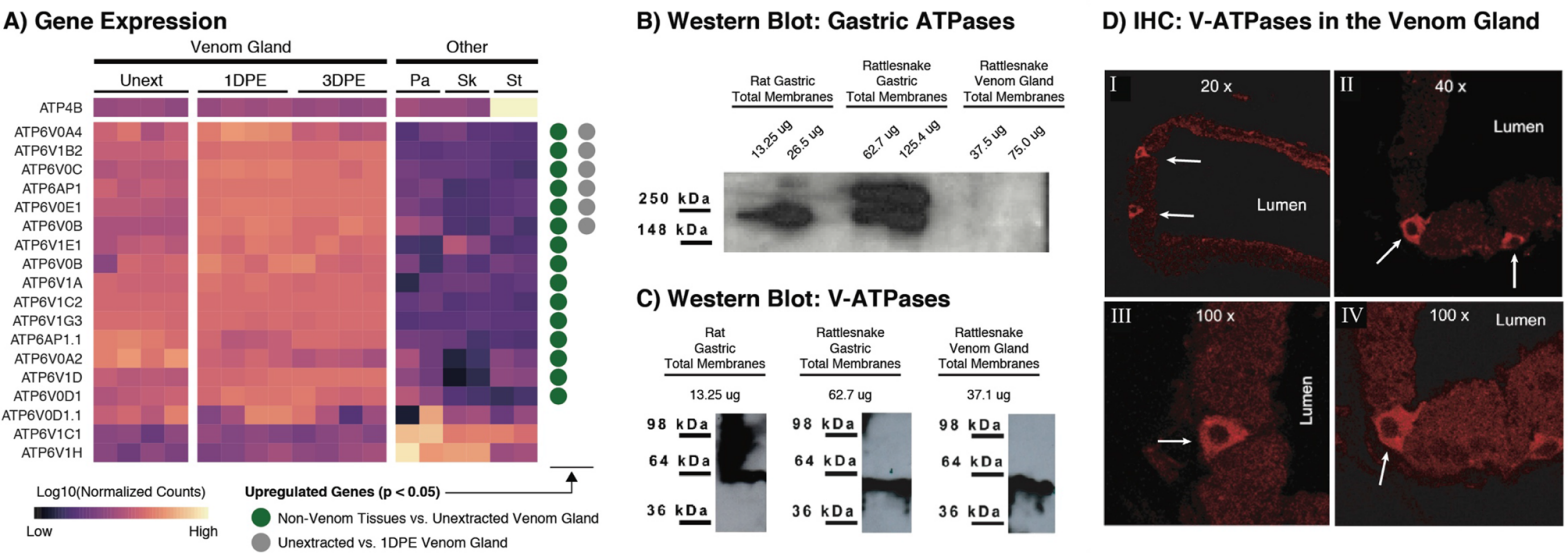
Vacuolar-ATPases are likely mechanisms driving venom gland acidification. (**A**) Gene expression heatmap of ATPase genes annotated with the “pH reduction” GO term, with circles on the right side indicating significant differential expression in the two focal pairwise comparisons. (**B,C**) Western blots testing for the presence of (**B**) gastric H+/K+ ATPases (cropped from a single gel and membrane) and (**C**) vacuolar ATPases in rattlesnake stomach and venom gland membranes (cropped from a single gel/membrane), with rat gastric membranes used as a positive control (cropped from a separate gel/membrane). No manipulations beyond cropping were applied to raw images. (**D**) Immunohistochemical staining showing an abundance of vacuolar-ATPases in mitochondria-rich cells in the venom gland epithelium (marked by arrows) at (I) 20×, (II) 40×, and (III–IV) 100× resolution.

While previous studies have demonstrated that acidification of the venom gland lumen plays an important role in venom storage, our initial analyses did not identify clear links to mechanisms associated with acidification beyond overrepresentation of the “phagosome acidification” term in analyses of the unextracted venom gland versus other secretory tissues (Fig. 3d). We performed additional post hoc characterization of 57 candidate genes annotated with the gene ontology term “pH reduction” (GO:0045851) to identify genes with potential roles in venom gland acidification. Of these candidate genes, 35 exhibited a detectable degree of expression in our gene expression dataset, 26 of these are significantly upregulated in the unextracted venom gland relative to non-venom body tissues, and six are upregulated between unextracted and 1DPE venom gland samples (Fig. 5A, Supplementary Information Figure 1). The majority of significantly upregulated genes are vacuolar-ATPases (V-ATPases), and *ATP6V1C2* in particular shows the greatest degree of upregulation in the unextracted venom gland relative to other secretory tissues (Fig. 5a). Notably, all six candidate genes with significant upregulation during venom production are components of V-ATPases (Fig. 5A, Supplementary Information Figure 1). In contrast, *ATP4B*, a major component of proton pumps driving gastric acid secretion, was not found to be upregulated in the venom gland relative to other secretory tissues, and instead shows high expression in the stomach only (Fig. 5A).

Western blots and immunohistochemical staining on total proteins extracted from Prairie Rattlesnake gastric and venom gland membranes were used to validate inferences of a role of V-ATPases in driving venom gland acidification (Fig. 5B–D). Western blots using polyclonal ATPAL1 antibody show a high prevalence of gastric H + /K + ATPases in rattlesnake gastric membrane only, with no detectable presence in the venom gland (Fig. 5B). Conversely, Western blots using αH56 antibody show prevalence of V-ATPases in both gastric and venom gland tissues (Fig. 5C), consistent with gene expression data, and immunohistochemical staining demonstrates that V-ATPases are concentrated in mitochondria-rich cells in the venom gland secretory epithelium (Fig. 5D).

## Discussion

This study provides new insight into the dynamic physiology of the snake venom gland and the molecular mechanisms associated with the secretory demands placed on this organ during venom production. We show that the depletion of venom in the rattlesnake venom gland is met with differential regulation of thousands of non-venom genes that orchestrate a complex suite of regulatory responses to support venom production, highlighting the complexity of this process. Our inferences provide the first detailed molecular characterization of this response, including regulatory mechanisms driving secretory function and epithelial maintenance, and highlight a role of cellular stress response activation during venom production. Characterization of the unextracted venom gland gene expression programs compared to other secretory tissues suggests unique activation of additional signaling mechanisms associated with cell growth, survival, and extreme secretory capacity, as well as further evidence for stress response activation, in the venom gland. Our results also provide new evidence for mechanisms of venom lumen acidification that maintains venom toxins in an inactive form during storage prior to envenomation.

Comparisons of gene expression between unextracted and 1 DPE venom gland tissues identified over 2500 non-venom genes that are differentially expressed within 24 h of venom depletion (Fig. 2a), emphasizing that venom production entails a complex and highly regulated process necessitating the activation of a large coordinated gene expression response to facilitate production of a comparatively small number of venom components. These genes are inferred to drive peripheral physiological and cellular processes that are likely central to cellular and physiological shifts necessary to facilitate venom production, including the upregulation of regulatory pathways, molecules and genes involved in protein production, transport, and export. This response also includes suites of genes associated with cellular stress response, pro-survival and cell cycle regulation, and cellular growth and proliferation signaling (Fig. 2). In particular, the high number and magnitude of inferences related to stress response activation imply an important balance between upregulated cellular activity to replenish venom stores rapidly and the mitigation of resulting cellular stress to prevent DNA damage, apoptosis, and cellular dysfunction. Activated cell growth and proliferation signaling may also be indicative of epithelial cell turnover and general epithelial maintenance, which would further compound cellular stress during venom production. Collectively, these findings indicate that the extreme performance of the venom gland during venom production necessitates the activation of stress response mechanisms, and that cellular stress may be an important and unappreciated constraint in the evolution and function of venom systems.

Evidence for the extreme physiological regulation of the venom gland during venom production raise questions about what broad physiological and regulatory mechanisms may distinguish the snake venom gland from other body tissues and secretory organs. Similar to analyses of venom production, inferences of pathway and regulatory molecule activation in the unextracted, steady-state venom gland relative to other secretory tissues include mechanisms of cellular stress response, and primarily those related to the UPR (Fig. 3b–d). We also find evidence for the activation of a largely discreet set of pathways and molecules broadly involved in cellular growth and proliferation, lipid metabolism, and cell cycle regulation compared to those activated during venom production (Fig. 3b,c), suggesting that a distinct suites of regulatory pathways drive general epithelial maintenance during this steady state. The PTEN signaling pathway, which typically acts to negatively regulate cell proliferation, was inferred to be activated to a relatively high degree in both analyses of venom production and the comparison between venom and non-venom tissues, suggesting that regardless of the state of venom gland activity, cell proliferation is monitored and controlled more so than in other secretory tissues.

Functional characterization of genes upregulated in the venom gland compared to other secretory tissues identified overrepresentation of terms related to cellular secretory function and machinery (i.e. related to endoplasmic reticulum, Golgi, vesicle transport, protein processing components or function) and responses to endoplasmic reticulum stress and unfolded proteins (Fig. 3d). Together with evidence of activated regulatory mechanisms, these findings appear to indicate a higher degree of cellular and secretory activity occurring in the unextracted venom gland than previously predicted^9^. Alternatively, evidence of heightened regulatory activity and cellular secretory function in the steady state venom gland may represent physiological adaptations of the gland to maintain a primed highly responsive steady state that facilitates venom production to respond rapidly to venom depletion.

All of our analyses emphasize an important role of stress response mechanisms in the snake venom gland. Notably, the venom gland exhibits a high baseline level of activated stress response mechanisms compared to other secretory tissues. This suggests that the storage of venom in the venom gland may induce elevated levels of cellular stress, and may additionally suggest that the venom gland ‘steady-state’ may actually involve active maintenance of venom stores via low constitutive levels of venom production, further evidenced by elevated venom gene expression in the unextracted gland (Fig. 1). While previous studies have found little evidence for turnover of venom components during storage prior to depletion^9,22–24^, and it is unclear to what extent observed mRNA expression corresponds to venom protein production, our results raise the possibility that a degree of venom production is constitutive and ongoing at all times.

Previous studies have shown that acidification of the venom gland lumen inhibits venom activity and thus plays an important role in self-protection against the venom activity during storage^9^. However, the mechanisms of venom gland acidification and how they relate to mechanisms of acidification in other tissues is unknown. In mammalian parietal cells, acidification is driven by hydrogen potassium ATPase proton pumps encoded by the genes *ATP4A* and *ATP4B*^25^, which act to secrete hydrochloric acid into the stomach lumen. In the rattlesnake venom gland, *ATP4B* exhibited high expression in the stomach as expected, but low expression in venom gland tissues, pancreas, and skin (Fig. 5), suggesting that hydrogen potassium ATPase pumps are not involved in venom gland acidification. Instead, analysis of candidate genes involved in proton transmembrane transport identified significant upregulation of multiple V-ATPases (Fig. 5) that, while typically associated with acidification of secretory vesicles^26^, drive luminal acidification in some tissues^27–30^. Western blots of rattlesnake stomach and venom gland membranes confirm the lack of gastric ATPases and abundance of V-ATPases in the venom gland, and immunohistochemical staining for V-ATPases indicate that V-ATPases are concentrated in mitochondria-rich cells present in the venom gland epithelium. Collectively, these findings strongly indicate a role of V-ATPases in the direct acidification of the venom gland lumen, and support previous inferences that mitochondria-rich cells in the venom gland epithelium are the primary drivers of gland acidification.

This study provides a valuable perspective on the complex nature of venom gland physiology in the Prairie Rattlesnake, and we expect that many aspects of our findings are likely applicable to snake venom gland physiology in general. However, the high degree of variation in venom phenotypes observed both among conspecific populations and between venomous snake species raises the question of whether there exists corresponding variation in specific aspects of venom gland physiology and function. Future studies that incorporate greater replication both within and across species will be vital to develop a comprehensive understanding of venom gland physiology, regulation, and evolution across the diversity of venomous snakes.

## Conclusion

Snake venom systems have emerged as an important model for addressing a broad array of biological, evolutionary and biomedical questions (e.g.^9,28–38^). Our analyses of the underlying physiological regulation of venom gland function provide new insight into the extreme cellular “environment” in which venom production takes place, highlighting that the venom gland itself may be as interesting a model system as the venoms it produces. Evidence for the broad coordination of physiological and cellular programs that accompany venom storage and production illustrate the complexity of regulatory systems that have co-evolved to enable venom gland function. These findings raise intriguing questions about co-evolution of these pathways with venom itself, and whether variation in venom phenotypes across species corresponds with nuanced differences in venom gland physiology and function. Our findings also raise the question of whether particular signaling responses related to venom gland physiological regulation are also coupled to regulation of venom—a process that remains poorly understood^1,3,8,11^.

Our analyses of the physiological functions of snake venom glands consistently emphasize a central role of stress response pathways in mediating cell stress and enabling extreme cellular performance. Beyond the venom gland, snakes are important models for other extreme physiological responses, including exceptional physiological upregulation, metabolic fluctuation, and organ regenerative growth upon feeding^39,40^. Intriguingly, previous studies of molecular mechanisms underlying this post-feeding regenerative growth in snakes have also suggested an important role of stress responses during extreme bouts of growth and regeneration, including those identified in this study (e.g., UPR, NRF2^39,40^). This raises the question of whether stress response activation may play a broadly important role in the evolution of diverse physiological adaptations in snakes.

## Supplementary information

Supplementary Information

## Data Availability

Gene expression data are available from the NCBI Short Read Archive at (ACCESSION PENDING). All relevant scripts and code are available at github.com/blairperry/VenomGlandPhysiol.
