# Supplementary material for: Physiological demands and signaling associated with snake venom production and storage illustrated by transcriptional analyses of venom glands
Source: Sci Rep. 2020 Oct 22;10:18083. doi: 10.1038/s41598-020-75048-y (PMC7582160; doi:10.1038/s41598-020-75048-y)
Supplement: Supplementary file 1 — Supplementary Information [file 41598_2020_75048_MOESM1_ESM.pdf]

Physiological demands and signaling associated with snake venom production and storage illustrated by transcriptional analyses of venom glands

Blair W. Perry[1], Drew R. Schield[1,2], Aundrea K. Westfall[1], Stephen P. Mackessy[3], & Todd A. Castoe[1, §]

[1]Department of Biology, 501 S. Nedderman Dr., The University of Texas Arlington, Arlington, TX, 76019, USA

[2]Department of Ecology and Evolutionary Biology, University of Colorado, Boulder, CO 80309, USA

[3]School of Biological Sciences, 501 20th Street, University of Northern Colorado, Greeley, CO 80639, USA

[§]**Author for Correspondence**: Todd A. Castoe, Department of Biology, University of Texas at Arlington, Arlington, TX, 76019, USA.

Email: todd.castoe@uta.edu *phone:* 817-272-9084 *fax:* 817-272-9615

Supplementary Methods

Generation of mRNA-seq data

Venom gland tissue samples were generated previously [1]. In brief, venom was manually expressed from one of the two venom glands of an adult male Prairie Rattlesnake, and the second venom gland was expressed 2 days later. One day later, the animal was humanely euthanized and venom gland tissues were dissected out and immediately snap frozen in liquid nitrogen. This process resulted in both a one day post-extraction (DPE) and three DPE venom gland tissue sample from the same animal. Unextracted venom gland, skin, pancreas, and stomach tissue was dissected from an additional male individual and frozen in liquid nitrogen. Total RNA was extracted from all tissue samples using Trizol Reagent (Invitrogen) and isopropanol. Four and two technical replicates were extracted for each venom gland and body-tissue treatment, respectively. Illumina mRNAseq libraries generated using poly-A selection and sequenced at Novogene on an Illumina NovaSeq platform using 150 bp paired-end reads.

mRNA-seq processing and pairwise analysis

Raw RNAseq data was quality-trimmed and filtered using Trimmomatic v0.33 [2] and mapped to the Prairie Rattlesnake reference genome using STAR v2.5.2b [3]. Raw read counts were generated with featureCounts v1.6.3 [4]. Count normalization and pairwise comparisons between unextracted and 1DPE venom gland, 1DPE and 3DPE venom gland, and body tissue and unextracted venom gland were conducted in DeSeq2 v1.26.0 [5], and resulting p-values were corrected using independent hypothesis weighting (IHW) using baseMean from DeSeq2 as the covariate [6]. Differentially expressed genes were defined as those with IHW p-value < 0.05.

Inferences of regulatory pathway ad molecular activity and analysis of overrepresented functional groups

Venom genes have been previously curated and annotated in the Prairie Rattlesnake reference genome [1], Annotated venom genes were excluded from subsequent analyses that focus on non-venom gene

regulation. Differentially expressed non-venom genes were then assigned an orthologous human gene identifier using orthology tables generated previously [7]. To infer broad patterns of regulatory pathway and molecule activity, differentially expressed genes were then analyzed using Core Analysis in Ingenuity Pathway Analysis (IPA) [8]. In the Core Analysis results, the following categories of canonical pathways were excluded: cancer, cardiovascular signaling, cellular immune response, disease-specific pathways, humoral immune response, Ingenuity Toxicity List Pathways, neurotransmitters and other nervous system signaling, pathogen-influenced signaling, and xenobiotic metabolism. Upstream regulatory molecule results were filtered by molecule type to include only genes, RNAs and proteins. Inferences of canonical pathway and upstream regulatory molecule activity with an overlap p-value < 0.05 and absolute activation z-score > 1 were considered significant.

To characterize functional groups of differentially expressed genes further, gene ontology (GO) analyses were performed specifically on sets of genes upregulated in the unextracted venom gland relative to non-venom tissues, and for those upregulated in 1DPE relative to unextracted venom gland tissues. GO terms with significant overrepresentation in these gene sets were determined using the ClueGO plugin v2.5.6 [9] for Cytoscape v3.7.2 [10] using a right-sided hypergeometric test of enrichment with default p-value correction, using all genes that met DeSeq2 input cutoffs. Terms with a corrected p-value < 0.05 were considered significantly enriched. Networks of enriched GO terms were further manually characterized and grouped based on similarity of function, tissue, or cellular process.

Mechanisms of venom gland acidification

To investigate potential mechanisms driving venom gland acidification, we first compared gene expression for a set of candidate genes annotated with the "pH reduction" GO term (GO: 0045851) to identify genes with evidence of informative upregulation in the venom gland relative to other secretory tissues and/or during venom production.

To validate inferences related to the roles of H+/K+ versus vacuolar ATPases in driving venom gland acidification, we performed Western immunoblot analyses and immunohistochemical staining of gastric and venom gland membranes. Stomach and venom gland tissues were dissected from an adult Prairie Rattlesnake, and epithelial cells were harvested after removal of connective tissue and fascia. Epithelial tissues were then minced on an ice-chilled glass plate prior to homogenization in 3 ml 10 mM PIPES/tris buffer pH 7.4 with 2mM ethylenediaminetetraacetic acid (EDTA) and 2 mM ethylene glycol-bis(2-aminoethylether)-N,N,N',N'-tetraacetic acid (EGTA) at 1500 rpm on ice. The remaining muscle and connective tissue was then removed by centrifugation at 3K rpm for 10 min at 4 ^{o}C. The resulting supernatant was layered onto 42% sucrose (w/v) in PIPES/tris buffer and overlaid with 5% sucrose. The samples were then centrifuged at 25K rpm for 90 min. at 4 ^{o}C in an SW28 swing rotor in a Beckman L8-70 ultracentrifuge. The membrane fraction, located at the interface of the 42 and 5% sucrose layers, was removed with a Pasteur pipette, transferred to a new centrifuge tube and topped with PIPES/tris buffer. Protein-containing membrane fractions were then pelleted by centrifuging at 34K rpm for 45 min at 4 ^{o}C. Following aspiration of the supernatant, the pellet was resuspended in tris/pipes buffer. Quantification of protein in membrane fractions was accomplished by a modified Lowry method utilizing the BCA Modified Lowry reagent from Promega (Madison, WI.). This material was then utilized (undiluted) for Western blots.

Thirty µl of each undiluted sample were run on a 7.5% acrylamide SDS-tricine reducing gel and transferred onto nitrocellulose membranes as described previously [11]. Non-specific binding was blocked by incubating membranes in 10 ml 5% nonfat milk in PBS-tween20 (20% w/v) for 30 min. The blots were then incubated in 10 ml blocking solution with 1:2000 (v/v) primary antibody for 1 hr at RT; ATPAL1 was at 5.0 µg/µl and αH56 was in 100% mouse serum. The polyclonal ATPAL1 (designed against a C-terminal epitope of the gastric H^+/K^+-ATPase) and αH56 (designed against the

56kDa subunit of the vacuolar H^+-ATPase) antibodies were used to test for the presence of H+/K+ ATPases and vacuolar ATPases, respectively, in gastric and venom gland membrane preparations [12,13]. Subsequently, the blots were washed for 3 x 15 min in PBS-tween and placed in 10 ml blocking solution with 1:20K secondary antibody. Following incubation at RT for 1 hr, the blots were again washed, incubated in 10 ml Supersignal West Pico chemiluminescent substrate solution (Pierce, Rockville, IL.) for 5 min at RT and exposed to high performance chemiluminescence film (Amersham International, Buckinghamshire, England).

Separately, main venom glands were fixed in 3.7% PBS-buffered formalin prior to being imbedded in paraffin wax and sectioned by microtome. The slides were dewaxed in xylene and rehydrated in an ethanol series, followed by PBS. To reduce background fluorescence during confocal microscopy, the antigen retrieval system (Dako Corp., Carpentaria, Ca.) was employed. The samples were then blocked with Dako Protein Block Serum and incubated overnight at 4 ^oC with 1:1000 mouse serum containing the αH56 polyclonal antibody. After washing 3x in 100 mM phosphate buffer (PB) pH 7.4, the secondary antibody (anti-mouse IgG conjugated to tetramethylrhodamine isothiocyanate (TRITC) in PB) was added to the samples and incubated for 1 hr in the dark at RT. The slides were then washed 3x in PB and mounted with Dako mounting medium. The labeled venom gland section was then visualized using a Zeiss LSM 550 confocal microscope with an excitation wavelength of 552 nm and long pass emission at 575 nm.

Supplementary Figures

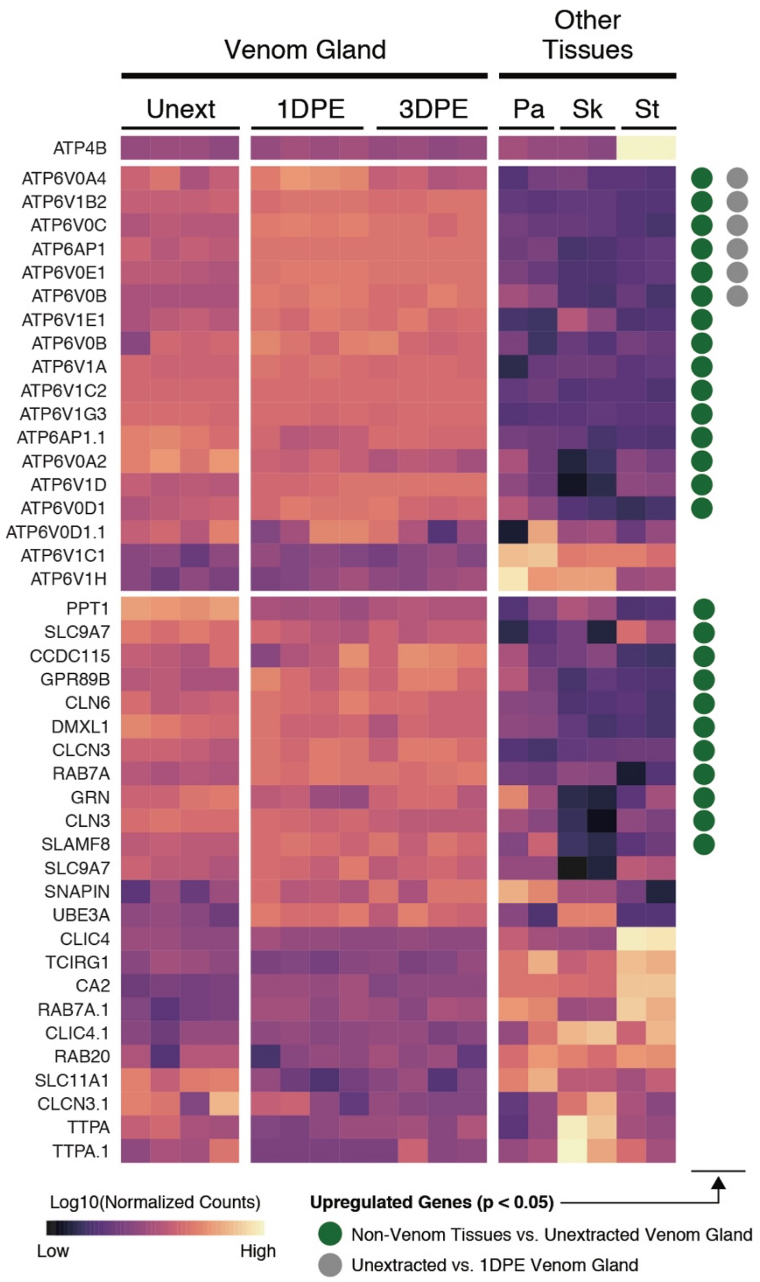

Supplementary Figure 1. Gene expression heatmap of full set of genes annotated with the "pH reduction" GO term, with circles on the right side indicating significant differential expression in the two focal pairwise comparisons.

1. Schield, D. R. *et al.* The origins and evolution of chromosomes, dosage compensation, and mechanisms underlying venom regulation in snakes. *Genome Res.* **29**, 590–601 (2019).

2. Bolger, A. M., Lohse, M. & Usadel, B. Trimmomatic: a flexible trimmer for Illumina sequence data. *Bioinformatics* **30**, 2114–2120 (2014).

3. Dobin, A. *et al.* STAR: ultrafast universal RNA-seq aligner. *Bioinformatics* **29**, 15–21 (2013).

4. Liao, Y., Smyth, G. K. & Shi, W. featureCounts: an efficient general purpose program for assigning sequence reads to genomic features. *Bioinformatics* **30**, 923–930 (2013).

5. Love, M. I., Huber, W. & Anders, S. Moderated estimation of fold change and dispersion for RNA-seq data with DESeq2. *Genome Biol.* **15**, 550 (2014).

6. Ignatiadis, N., Klaus, B., Zaugg, J. B. & Huber, W. Data-driven hypothesis weighting increases detection power in genome-scale multiple testing. *Nat. Methods* **13**, 577 (2016).

7. Perry, B. W. *et al.* Molecular adaptations for sensing and securing prey and insight into amniote genome diversity from the garter snake genome. *Genome Biol. Evol.* **10**, 2110–2129 (2018).

8. Krämer, A., Green, J., Pollard Jr, J. & Tugendreich, S. Causal analysis approaches in ingenuity pathway analysis. *Bioinformatics* **30**, 523–530 (2013).

9. Bindea, G. *et al.* ClueGO: a Cytoscape plug-in to decipher functionally grouped gene ontology and pathway annotation networks. *Bioinformatics* **25**, 1091–1093 (2009).

10. Shannon, P. *et al.* Cytoscape: a software environment for integrated models of biomolecular interaction networks. *Genome Res.* **13**, 2498–2504 (2003).

11. Smith, C. F. & Mackessy, S. P. The effects of hybridization on divergent venom phenotypes: characterization of venom from Crotalus scutulatus scutulatus× Crotalus oreganus helleri hybrids. *Toxicon* **120**, 110–123 (2016).

12. Granger, D., Marsolais, M., Burry, J. & Laprade, R. V-type H+-ATPase in the human eccrine sweat duct: immunolocalization and functional demonstration. *Am. J. Physiol. Physiol.* **282**, C1454–C1460 (2002).

13. Mercier, F. *et al.* Antibody epitope mapping of the gastric H+/K+-ATPase. *Biochim. Biophys. Acta (BBA)-Biomembranes* **1149**, 151–165 (1993).
